# B7-H4 Pathway in Islet Transplantation and **β**-Cell Replacement Therapies

**DOI:** 10.1155/2011/418902

**Published:** 2011-10-13

**Authors:** Xiaojie Wang, Jianqiang Hao, Daniel L. Metzger, Ziliang Ao, Mark Meloche, C. Bruce Verchere, Lieping Chen, Dawei Ou, Alice Mui, Garth L. Warnock

**Affiliations:** ^1^Department of Surgery, University of British Columbia, Vancouver, BC, Canada V5Z 4E3; ^2^Department of Paediatrics, University of British Columbia, Vancouver, BC, Canada V6H 3V4; ^3^Department of Pathology and Laboratory Medicine, University of British Columbia, Vancouver, BC, Canada V5Z 4H4; ^4^Department of Immunobiology, Yale University School of Medicine, New Haven, CT 06519, USA

## Abstract

Type 1 diabetes (T1D) is a chronic autoimmune disease and characterized by absolute insulin deficiency. *β*-cell replacement by islet cell transplantation has been established as a feasible treatment option for T1D. The two main obstacles after islet transplantation are alloreactive T-cell-mediated graft rejection and recurrence of autoimmune diabetes mellitus in recipients. T cells play a central role in determining the outcome of both autoimmune responses and allograft survival. B7-H4, a newly identified B7 homolog, plays a key role in maintaining T-cell homeostasis by reducing T-cell proliferation and cytokine production. The relationship between B7-H4 and allograft survival/autoimmunity has been investigated recently in both islet transplantation and the nonobese diabetic (NOD) mouse models. B7-H4 protects allograft survival and generates donor-specific tolerance. It also prevents the development of autoimmune diabetes. More importantly, B7-H4 plays an indispensable role in alloimmunity in the absence of the classic CD28/CTLA-4 : B7 pathway, suggesting a synergistic/additive effect with other agents such as CTLA-4 on inhibition of unwanted immune responses.

## 1. Introduction

Type 1 diabetes (T1D) is fatal unless treated with insulin. Injection of insulin prevents the hyperglycemic complications of T1D, including ketoacidosis and coma. However, exogenous administration of excessive or inadequate amounts of insulin often results in hypo- and hyperglycemia, respectively [1–3]. Combination of intensive glycemic monitoring and best medical therapy provides better control of insulin level and reduces the microvascular and macrovascular complications of diabetes, but it also increases the risk of severe hypoglycemia [1–3]. In selected patients, islet transplantation is a reasonable therapeutic option. Restoration of *β*-cell mass by whole-pancreas or islet cell transplantation provides physiologically regulated insulin as well as other hormones, such as glucagon to avoid life-threatening unregulated glucose levels [4–6]. Whole-pancreas transplantation is a major surgical procedure and is usually performed in conjunction with a kidney transplant, either simultaneous pancreas-kidney transplant (SPK) or pancreas-after-kidney transplant (PAK), for patients with end-stage renal disease. By contrast, islet cell transplantation is a less invasive and relatively simple procedure. Isolated islets are infused into the liver through the portal vein, guided by fluoroscopic cannulation. The one-year success rate is comparable for both types of transplantations. Significant progress has been made in islet transplantation, especially the establishment of the “Edmonton protocol” in 2000 [7]. This success of this protocol is based on provision of both sufficient islet-cell mass and a steroid-free immunosuppressive regimen. However, the subsequent followups conducted worldwide in several centers show a constant decline in graft function [8]. Despite this, the majority of recipients benefit from reduced overall insulin requirements; improved C-peptide secretion and HbA1C levels; decreased development of microvascular complications; and fewer complications related to episodes of hypoglycemia after 5-year followup [9]. Therefore, islet transplantation is superior to best medical therapy in terms of better control of metabolism and prevention of devastating hypoglycemia over long periods.

One of the two major challenges in islet transplantation is the limited supply of donor islets. The strategies for expanding donor islet supply include the construction of insulin-producing cells *de novo.* This can be achieved through four different ways: (1) from transdifferentiation of other types of cells, such as hepatic cells; (2) from differentiation of pancreatic progenitor cells, such as acinar or ductal cells; (3) from differentiation of pluripotent stem cells, such as embryonic stem cells; and (4) from expansion of existing *β* cells. There are some excellent reviews on this field, and we will not address this issue in detail [10, 11].

The other central barrier for islet transplant success is graft failure, or so- called rejection. The current glucocorticoid-free immunosuppressive regimen for islet transplantation includes tacrolimus (FK506) and either sirolimus (rapamycin) or mycophenolate mofetil (MMF). Although these immunosuppressive drugs control acute rejection and enhance islet allograft survival, lack of long-term efficacy/insulin independence and immunosuppressant-associated side effects (including risks of cancer, infection, nephrotoxicity, cardiovascular-related diseases, and even direct islet toxicity) hamper this great application. The existing data reveal that current immunosuppressive drugs induce cytotoxicity to islets and reduce *β*-cell function. The function of human islets is impaired by chronic exposure to FK506 or MMF [12]. FK506, but not MMF, damages human islet graft function in diabetic NOD.SCID mice [12]. All three drugs (FK506, MMF, and rapamycin) increase apoptosis in islets [12]. Therefore, new strategies are needed to avoid generalized suppression of immunity and its associated cytotoxicity without alteration of *β*-cell function. All types of immunosuppressive drugs are presumed to have some degree of nonspecific toxicity. In this regard, islet graft function should be better maintained in the absence of long-term ongoing immunosuppression. Ideally, a recipient's immune system would not reject an islet graft, while continuing to respond to all other foreign antigens normally. Therefore, an ultimate goal in transplantation is to induce antigen-specific tolerance.

## 2. Mechanisms of Rejection of Transplanted Grafts

Rejection is a normal adaptive immune reaction to foreign antigens. The discovery of the major histocompatibility complex (MHC) by Jean Dausset in 1958 established human leukocyte antigen (HLA) as a transplant antigen. Transplantation rejection occurs between individuals with mismatched HLA haplotypes. The degree of rejection is determined by the level of mismatch in the MHC haplotypes. The more closely MHC haplotypes are matched between donor and host, the greater degree of acceptance is observed [13, 14]. In this regard, MHC molecules play a primary role in graft rejection. Rejection of donor grafts is the result of humoral and cell-mediated reactions to major MHC antigens, known as HLA in humans, H2 in mice, and RT1 in rats [13]. Recognition of foreign antigens presented on MHC molecules by T-cell receptors (TCR) initiates T-cell-mediated immune responses that result in the destruction of transplanted grafts.

T cells play a central role in allograft rejection. Direct evidence from adoptive transfer experiments shows that allograft immunity is conferred after adoptive transfer of lymphocytes, but not of serum [15, 16]. In concordance with this function, depletion of T cells by injection of antibody results in acceptance of mouse islet allografts [17, 18], suggesting that T cells determine the fate of transplantation. Subsequent experiments revealed that CD4^+^ but not CD8^+^ subsets are necessary for the rejection of islet allografts [19].

**Figure 1:**
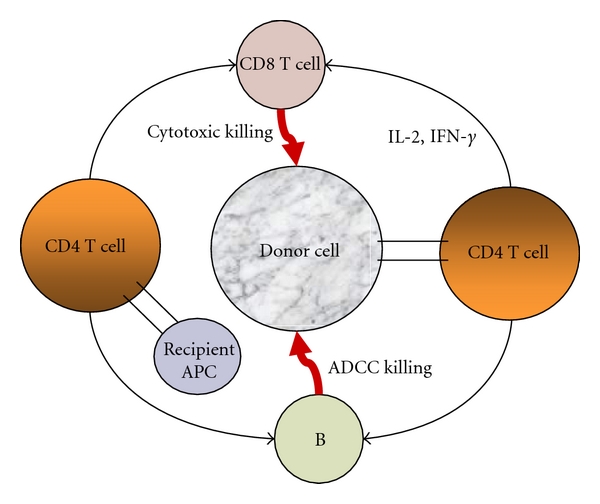
Mechanism of graft rejection. Allogeneic T cells recognize antigen through either a direct or an indirect pathway. In the direct pathway, T cells recognize intact allogeneic antigen on the surface of donor-derived APCs. This pathway is thought to predominate in acute rejection. In the indirect pathway, recipient APCs process donor-derived alloantigen into peptides and then present them to recipient T cells. This pathway is thought to dominate chronic rejection. The activation of T cells through either pathway results in killing the donor graft.

T cells recognize alloantigens through two distinct pathways. An alloantigen can be presented on either donor (direct pathway) or recipient (indirect pathway) MHC molecules (Figure 1). In either pathway, interaction of CD4^+^ T cells with an alloantigen results in activation and production of cytokines such as IFN-*γ* and interleukin- (IL-) 2 that promote differentiation and proliferation of cytotoxic CD8^+^ T cells, macrophages, and B cells. These alloreactive cells can lyse transplanted grafts or produce cytokines that induce necrosis of donor tissues (Figure 1).

CD4^+^ cells, also called helper T cells (Th), play a dominant role in initiating graft rejection [14, 16]. CD4^+^ Th cells can differentiate into one of 4 subtypes. Transcription factors T-bet, GATA-3, forkhead box P3 (FoxP3), and the retinoic acid receptor-related orphan receptor *γ* (ROR*γ*t) direct differentiation of Th1, Th2, Treg, and Th17, respectively. Once differentiated, each lineage secretes a specific cytokine profile. For example, Th1 and Th2 subsets secrete interferon *γ* (IFN-*γ*) and IL-4/IL-10, respectively [14]. The Th1, Th2, Treg, and Th17 subsets cooperate and influence the outcome of transplanted grafts. The Th1 cytokine profile was previously thought to be associated with allograft damage and rejection, while the Th2 profile favors the acquisition of protection and tolerance [20]. However, this simplified Th1/Th2 paradigm may not be sufficient to explain redundant effects of cytokine networks on the outcome of transplantation *in vivo*.

The newly characterized regulatory T-cell subset, Tregs, plays an anti-inflammatory role and maintains tolerance to self-antigens. Tregs (CD4^+^ CD25^+^) suppress proliferation of CD4^+^ CD25^−^ T cells, CD8^+^ T cells, dendritic cells, B cells, macrophages, mast cells, osteoblasts, NK, and NKT cells in an antigen-non-specific manner [21]. Tregs play an important role in preventing transplant rejection and generating tolerance [22].

## 3. Tregs and Transplantation

Treg-mediated tolerance induction has been well established as a nondeletional strategy to modulate alloreactive T-cell responses. The role of Tregs in maintaining self-tolerance was described by Sakaguchi's group, which showed that Tregs from the thymus protect the host from autoimmune diseases [23].

Tregs can be categorized into two main types (naturally occurring Tregs, nTregs, and inducible Tregs, iTregs) based on their origins, mechanisms, and modes of action [24]. They are both called regulatory T cells because they have similarities in terms of phenotype and function. First, both express Foxp3 and suppress the proliferation of effector cells. Secondly, Tregs will be absent from the thymus or the periphery, if proximal TCR signaling is disrupted, either through genetic manipulation or the use of calcineurin inhibitors. Thirdly, the development of both types of Tregs is dependent on cytokines such as IL-2. The two subsets of Tregs can be differentiated by their distinct suppressive mechanisms and other characteristics. nTregs develop in the thymus and constitute approximately 5–10% of peripheral CD4^+^ T cells. CD4^+^CD25^+^ Tregs suppress effector T-cell proliferation *in vitro* through a cell contact-dependent mechanism, and their function is cytokine-independent [24]. A role for nTregs in the development of transplantation tolerance was first indicated by their ability to suppress mouse GVHD following adoptive transfer [25].

The second population of Treg subsets (iTregs) is distinct from nTregs and arises during immune responses in the periphery. iTregs suppress immune responses through secretion of immunosuppressive cytokines. Th3 and Tr1 cells induce suppression through secretion of TGF-*β* and IL-10, respectively, [24]. Tr1 was first identified by Groux et al. [26]. Naïve T cells from ovalbumin (OVA) TCR-transgenic mice stimulated with OVA and IL-10 suppress antigen-specific activation *in vitro* and prevent the development of colitis *in vivo* [26]. Moreover, the supernatant from Tr1 strongly suppresses alloantigen-specific T-cell proliferation [27]. Tr1 cells tend to migrate toward the site of inflammation. Tregs can be detected in the extralymphoid sites. It shows that CD4^+^CD25^+^ Tregs are overexpressed within tolerated allograft [28]. Th3 was originally described in oral tolerance in mice induced by myelin basic protein (MBP) [29]. Th3 suppressive cells may be converted from nonregulatory cells in the presence of TGF-*β* [30]. The existence of suppressive cells of nonthymus origin was confirmed by the demonstration of CD4^+^CD25^+^ conversion from CD25^−^ precursors in thymectomized mice, and that these non-thymus-derived Tregs can suppress skin allograft rejection [31].

## 4. Costimulation Blockade in Transplantation

**Figure 2:**
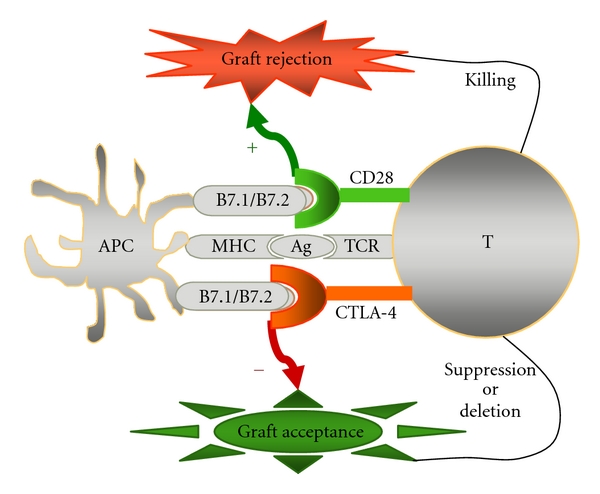
The outcome of transplantation is determined by cosignalling. Full T-cell activation requires two signals. The first is antigen-specific recognition of TCR and alloantigen presented on MHC. The second is costimulation which can be positive or negative. In the presence of positive cosignalling (such as with CD28), T-cell proliferation occurs. These activated T cells attack the donor graft and results in graft rejection. One the other hand, the activated T-cell response is terminated in the presence of negative co-signalling (such as with CTLA-4) which protects the graft from destruction.

T cells are the principal mediator of alloreactive reactions, and their full activation requires 2 signals. The first is provided by the interaction of antigen-specific TCRs and their cognate alloantigens presented on MHCs. The second signal is antigen nonspecific. It can be provided *in trans* by APCs and requires cell-cell contact [32]. Signal 1 alone results in no response. Signals 1 plus 2 lead to either activation or inhibition of the T-cell response, depending on which cosignal pathway dominates. In the presence of positive signals, such as CD28, T cells become activated upon stimulation with foreign antigen. On the contrary, activated T-cell proliferation is terminated with negative coinhibitory signals, such as CTLA-4 [33]. The requirement of two separate signals for full activation of the intracellular signalling cascade, IL-2 transcription, T-cell proliferation, and effector function suggests a critical role of cosignalling pathways in determining the fate of transplantation (Figure 2). Much attention has been focused on using agents which are either coinhibitory with negative-signal molecules, or antagonistic to positive-signal molecules, to control allograft rejection.

The discovery of the requirement of both recognition signal (signal 1) and verification signal (signal 2) for effective T-cell activation has elucidated potential targets for immunosuppression that are highly T-cell specific. In particular, the need for additional cosignalling to avoid anergy or apoptosis has generated the strategy for tolerance induction using costimulation blockade. In fact, T-cell clones cultured with antigen and MHC alone lead to unresponsiveness [34]; in other words, T cells will undergo anergy or apoptosis without signal 2. This observation suggests the attractive prospect that allografts could become tolerated if signal 2 is blocked. Although diverse strategies have been investigated to enhance graft survival in terms of improved efficacy and reduced toxicity, different degrees of side effects are unavoidable. Therefore, tolerance induction or withdrawal of long-term usage of immunosuppressants is preferred. Theoretically, it may be achieved by using costimulation blockade.

## 5. B7-H4 Coinhibitory Pathway

B7-H4 (B7x or B7S) was identified by three independent groups using expressed sequence tags (EST) with homology to the B7 family [35–37]. The genes for human and mouse B7-H4 are located on chromosomes 1 and 3, respectively. Genomic DNA of B7-H4 consists of 6 exons and 5 introns, and mature protein is encoded by an 849-bp region spanning exons III, IV, and part of V. In both genes, exons I and II form a signal peptide, and V encodes transmembrane and intracellular regions.

The extracellular region of B7-H4 contains one Ig V and one Ig C domain. Within the extracellular domain, mouse B7-H4 shares 90% and 99% amino acid (aa) sequence identity with human and rat B7-H4, respectively, suggesting a conserved identity among species. Although it shares only 21–29% aa sequence identity with B7.1, B7.2, B7-H1, B7-H2, B7-H3, and PD-L2 [35], B7-H4 exhibits a similar overall structure with other members in the B7 family.

B7-H4 mRNA is ubiquitously expressed in both lymphoid and nonlymphoid tissues, including placenta, kidney, liver, lung, ovary, testis, and spleen, suggesting that it plays a potential role in peripheral tissues [35–37]. The mature protein is a 50- to -80-kDa glycosylated molecule with a 28-kDa protein core, and its expression appears to be restricted, suggesting posttranscriptional/translational regulation. B7-H4 expression is induced on mitogen- or LPS-activated B cells, T cells, dendritic cells, monocytes, and macrophages [35–37].

The Ig V region is responsible for the binding with the counter-receptor. In B7.1 and B7.2, the V region contains a conserved strand of A′GFCC′C for CTLA-4/CD28 binding. B7-H4 contains no such conserved face. In fact, B7-H4 binds to a receptor on activated T cells but not to CTLA-4, ICOS, or PD-1, according to FACS analysis of a B7-H4-transfected 293 cell line [35–37]. B7-H4 is expressed on wild type (WT) but not on B- and T-lymphocyte- attenuator- (BTLA-) deficient cells, suggesting a possible role of BTLA as a B7-H4 receptor [37]. However, recent studies have indicated that BTLA does not bind to B7-H4 directly, and that herpes virus entry mediator (HVEM) may be the unique BTLA ligand [38]. The receptor for B7-H4 is still unknown.

The primary function of B7-H4 appears to downregulate the T-cell response. Several lines of evidence from both *in vitro* and *in vivo* data support this notion. B7-H4 inhibits normal mouse and human TCR-induced T-cell proliferation, cytokine production, and cytotoxicity *in vitro *[35–37]. Similarly, the proliferation of T cells and the secretion of IFN-*γ* activated by autoantigens (insulin, GAD, and IA-2) from human T1D patients were suppressed in the presence of B7-H4. In addition, ectopic expression of B7-H4 in human *β* cells also protects these cells from cytotoxicity induced by *β*-cell antigen-specific T-cell clones derived from T1D patients [39]. The negative effects of B7-H4 on T cells are also confirmed in several *in vivo* systems. Administration of B7-H4.Ig impairs CTL activity in a mouse graft-versus-host disease (GVHD) model [35]. In concordance with this inhibitory effect, administration of monoclonal antibody that blocks endogenous B7-H4 expression increases T-cell proliferation and IL-2 production [35]. Similarly, the injection of a blocking mAb against B7-H4 promotes T-cell responses and exacerbates experimental autoimmune encephalomyelitis (EAE) [36]. Collectively, these results suggest that B7-H4 is a novel negative regulator in the B7 family.

It is not clearly understood how B7-H4 affects the immune responses. Studies into the mechanisms by which B7-H4 regulates T-cell immunity show that the suppressive activity of APCs is associated with expression of B7-H4 *in vitro* [40]. B7-H4 expression on APCs is triggered by Tregs through production of IL-10. Blockade of B7-H4 reduces the suppressive activity mediated by Treg conditioned APCs. In addition, Tregs exhibit suppressive activity by stimulating B7-H4 expression through IL-10. Furthermore, the high expression level of B7-H4 in various cancer cells suggests that it may help those tumors escape immune surveillance [41]. Primary ovarian tumour cells express intracellular B7-H4, whereas only a fraction of tumour macrophages express surface B7-H4. B7-H4^+^ tumour macrophages, but not primary ovarian tumour cells, suppress tumour-associated antigen-specific T-cell immunity. IL-6 and IL-10 are highly expressed in the tumour environment, and they trigger macrophage B7-H4 expression [42]. Collectively, these results suggest that a collaborative interaction between B7-H4 and Tregs may downregulate T-cell immunity.

B7-H4 deficient mice display normal responses to several types of airway inflammation, and they show augmented Th1 responses, suggesting a preferential regulation on Th1 subsets [43]. Interestingly, a subsequent study revealed that B7-H4 suppresses neutrophil-mediated immune responses, indicating its role in innate immunity [44].

## 6. B7-H4 and Transplantation

As described earlier, B7-H4 inhibits alloreactive CTL activity in mouse GVHD and extends the survival of mice with GVHD [35]. The role of B7-H4 in islet transplantation was first investigated by our group [45]. Local expression of B7-H4 by a recombinant adenovirus (Ad-B7-H4) promotes islet allograft survival in a fully MHC-mismatched mouse model (from BALB/c to C56BL/6) [45]. This result has subsequently been confirmed by Yuan's group, using administration of a B7-H4-transfected NIT cell line into diabetic C57BL/6 mice [46]. We showed that survival is associated with reduced CD8^+^ T cells, preserved *β*-cell function, and upregulation of Foxp3^+^ in the allograft. Yuan's group showed a reduced amount of IFN-*γ* and increased Tregs in the spleen of B7-H4-treated recipients. Moreover, we used a secondary transplantation model to demonstrate that B7-H4 not only promotes allograft survival but also induces donor-specific tolerance [45, 47].

Alloreactive responses can be modulated through either deletional or nondeletional mechanisms. B7-H4 controls alloreactive responses through downregulating cytotoxic CD8^+^ subsets in the early stage, suggesting a deletional mechanism in tolerance induction [45]. Furthermore, transcription of IFN-*γ* and granzyme B were significantly decreased in Ad-B7-H4—treated allografts, suggesting that B7-H4 preferentially inhibits Th1 and CTL locally [47]. The inhibition of Th1 response by B7-H4 is also demonstrated in B7-H4-deficient mice which exhibit augmented expression level of both IFN-*γ* and T-bet in response to *Leishmania major* infection [43]. Whether a reduced amount of CD8^+^ is associated with Th1-related cytokines, or that low levels of IFN-*γ* results in a reduced CD8 population has not been investigated. This finding demonstrates that functional allograft is preserved by B7-H4-mediated negative co-signalling which limits both helper CD4^+^ cells towards Th1 formation and cytotoxic CD8^+^ cell towards killing donor tissues.

Apart from deletion-mediated tolerance induction, suppression can also be considered a nondeletional mechanism to modulate alloreactive T-cell responses. In the long term, B7-H4 preferentially upregulates the number of Foxp3 in the allograft and Tregs in the periphery, suggesting the involvement of a suppressive mechanism for allograft survival [45, 47]. The outcome of the alloreactive immune response is determined by the balance of effector and regulatory T cells in the process of priming naïve CD4^+^ T cells in response to alloantigen stimulation. The development of Th1/Th2 effectors often dominates alloreactive T-cell responses, whereas the conversion of Foxp3^+^ from naïve or effector T cells occurs only in the presence of specific stimuli. Studies into the relationship between helper and regulatory T-cell subsets reveal that the development of Foxp3^+^ in the periphery is profoundly inhibited by Th1/Th2 activities [48]. Cytokines for Th1/Th2 polarization, such as IFN-*γ*, IL-12, and IL-4, inhibit Treg differentiation from naïve cells induced by TGF-*β*1, whereas blocking of IFN-*γ* and/or IL-4 could promote Foxp3^+^ Treg differentiation both *in vitro* and* in vivo *[48]. Improved allograft survival in B7-H4-treated recipients may be due to the well-controlled alloreactive T-cell proliferation suppressed by increased number of Foxp3^+^. Reduced IFN-*γ* in the early stage may facilitate the conversion of Foxp3^+^ from effector or naïve T cells in the late stage.

**Figure 3:**
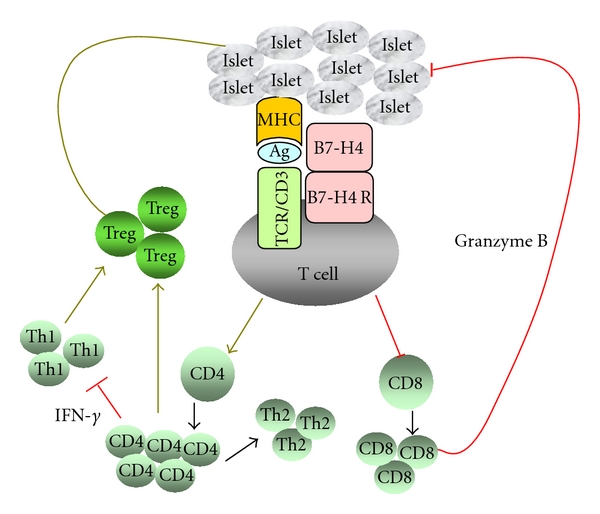
The mechanism of B7-H4 action in islet transplantation. Interaction between B7-H4 and an unknown receptor on activated T cells leads to a series of intracellular signalling cascades that result in improved allograft survival. B7-H4 protects allograft rejection through deletional and nondeletional mechanisms. It can limit cytotoxic CD8^+^ proliferation and transcription of granzyme B. It also reduces the Th1 response which may facilitate generation of suppressive Foxp3^+^ Tregs. The combination of controlling activated T-cell proliferation and promoting regulatory T-cell subsets results in enhanced allograft survival and induction of donor-specific tolerance.

B7-H4 enhances islet allograft survival and induces donor-specific tolerance through deletional and non-deletional mechanisms (Figure 3). However, the mechanism for tolerance induction and maintenance is not fully understood. A similar number of Tregs in the periphery of failed and surviving recipients treated with B7-H4 after second-set transplants demonstrated that factors other than Tregs may contribute to tolerance maintenance, such as inefficient inhibition of memory T cells generated in second-set transplants [47]. Theoretically, iTregs are supposed to be donor-specific because they are converted from effector cells that share a similar TCR repertoire. Therefore, the generation of iTregs might result in donor-specific tolerance. Epigenetic studies reveal that CpG dinucleotides at the Foxp3 locus are methylated in naïve CD4^+^CD25^−^, activated CD4^+^ T cells, and TGF-*β*-induced iTregs but completely demethylated in nTregs, demonstrating closely compact nucleosomes in the formal subsets that prevent transcription [49]. In fact, iTregs are particularly unstable and tend to lose Foxp3 expression more easily than nTregs. In B7-H4-treated recipients, suppressive Tregs and cytopathogenic Teffs can be reprogrammed upon secondary donor-specific antigen stimulation, indicating that Tregs are involved in tolerance induction but play a minimal role in tolerance maintenance. The relative contributions of nTregs and iTregs in terms of synergism in promoting tolerance or redundant remain to be elucidated.

## 7. B7-H4 and Autoimmunity

Mice treated with B7-H4-neutralizing mAb that blocks endogenous B7-H4 expression develop accelerated and much more robust EAE, demonstrating that B7-H4 downregulates autoimmune EAE [36]. Moreover, regulation of myelin oligodendrocyte glycoprotein- (MOG-) induced EAE by B7-H4-blocking mAb is associated with increased CD4^+^, CD8^+^, and CD11b^+^ macrophages, suggesting its role in modulating the interaction between T cells and APCs in EAE [36].

Our unpublished data show that early treatment of NOD mice with B7-H4.Ig fusion protein significantly reduces the incidence of spontaneous autoimmune disease to 28.6%, compared to 67.8% in controls, suggesting its efficacy in preventing the destruction of insulin-producing *β* cells by autoreactive T cells. These promising data demonstrate B7-H4's potential role in inhibiting recurrence of autoimmune diabetes in islet transplantation.

## 8. Future Directions and Conclusions

Much progress has been made in improving allograft survival and induction of tolerance using costimulation blockade. CTLA-4.Ig prolongs survival of allograft and xenografts in various rodent models [50–52]. Its mutant form LEA29A (belatacept), which increases the efficacy of inhibition through enhanced binding capacity and decreased dissociation rate with B7.1/B7.2, shows promising results in nonhuman primates and in human renal transplantation and is currently in phase III clinical trials [53, 54]. Programmed cell death ligand 1 (PD-L1) fusion protein, PD-L1.Ig, prolongs cardiac allograft survival in CD28-deficient but not in wild-type recipients, suggesting that PD-1:PD-L1 and CD28/CTLA-4:B7 may be two distinct pathways in alloimmunity [55]. Combination of PDL1.Ig and anti-CD154 prolongs islet allograft survival [56].

In a mouse solid organ (heart) transplantation model, B7-H4 plays a nonredundant regulatory role and functions dominantly in the absence of CD28/CTLA-4:B7 signals, suggesting its synergistic effects with CTLA-4 to control allograft rejection, especially facilitatory to tolerance induction [57]. In order to design new strategies for preventing both alloreactive and autoreactive immune responses and generating donor-specific tolerance, it will be important to identify the detailed mechanisms in each cosignalling pathway.

## Abbreviations

APC: Antigen- presenting cell
TCR: T-cell receptor
EST: Expressed sequence tagged
MHC: Major histocompatibility complex
HLA: Human leukocyte antigen
HbA1C: Hemoglobin A1C
IL: Interleukin
OVA: Ovalbumin.
